# Striatal Afferent BDNF Is Disrupted by Synucleinopathy and Partially Restored by STN DBS

**DOI:** 10.1523/JNEUROSCI.1952-20.2020

**Published:** 2021-03-03

**Authors:** Kathryn M. Miller, Joseph R. Patterson, Joseph Kochmanski, Christopher J. Kemp, Anna C. Stoll, Christopher U. Onyekpe, Allyson Cole-Strauss, Kathy Steece-Collier, Jacob W. Howe, Kelvin C. Luk, Caryl E. Sortwell

**Affiliations:** ^1^Department of Translational Neuroscience, College of Human Medicine, Michigan State University, Grand Rapids, Michigan 49503; ^2^Neuroscience Graduate Program, College of Natural Science, Michigan State University, East Lansing, Michigan 48824; ^3^Department of Pharmacology and Toxicology, Michigan State University, East Lansing, Michigan 48824; ^4^Center for Neurodegenerative Disease Research, Department of Pathology and Laboratory Medicine, University of Pennsylvania Perelman School of Medicine, Philadelphia, Pennsylvania 19104; ^5^Hauenstein Neuroscience Center, Mercy Health Saint Mary's, Grand Rapids, Michigan 49503

**Keywords:** α-synuclein, brain-derived neurotrophic factor, deep brain stimulation, neuroprotection, preformed fibril

## Abstract

Preclinical studies show a link between subthalamic nucleus (STN) deep brain stimulation (DBS) and neuroprotection of nigrostriatal dopamine (DA) neurons, potentially through brain-derived neurotrophic factor (BDNF) signaling. However, the question of whether DBS of the STN can be disease-modifying in Parkinson's disease (PD) remains unanswered. In particular, the impact of STN DBS on α-synuclein (α-syn) aggregation, inclusion-associated neuroinflammation, and BDNF levels has yet to be examined in the context of synucleinopathy. To address this, we examined the effects of STN DBS on BDNF using the α-syn preformed fibril (PFF) model in male rats. While PFF injection resulted in accumulation of phosphorylated α-syn (pSyn) inclusions in the substantia nigra pars compacta (SNpc) and cortical areas, STN DBS did not impact PFF-induced accumulation of pSyn inclusions in the SNpc. In addition, nigral pSyn inclusions were associated with increased microgliosis and astrogliosis; however, the magnitude of these processes was not altered by STN DBS. Total BDNF protein was not impacted by pSyn inclusions, but the normally positive association of nigrostriatal and corticostriatal BDNF was reversed in rats with PFF-induced nigrostriatal and corticostriatal inclusions. Despite this, rats receiving both STN DBS and PFF injection showed increased BDNF protein in the striatum, which partially restored the normal corticostriatal relationship. Our results suggest that pathologic α-syn inclusions disrupt anterograde BDNF transport within nigrostriatal and corticostriatal circuitry. Further, STN DBS has the potential to exert protective effects by modifying the long-term neurodegenerative consequences of synucleinopathy.

**SIGNIFICANCE STATEMENT** An increase in brain-derived neurotrophic factor (BDNF) has been linked to the neuroprotection elicited by subthalamic nucleus (STN) deep brain stimulation (DBS) in neurotoxicant models of Parkinson's disease (PD). However, whether STN DBS can similarly increase BDNF in nigrostriatal and corticostriatal circuitry in the presence of α-synuclein (α-syn) inclusions has not been examined. We examined the impact of STN DBS on rats in which accumulation of α-syn inclusions is induced by injection of α-syn preformed fibrils (PFFs). STN DBS significantly increased striatal BDNF protein in rats seeded with α-syn inclusions and partially restored the normal corticostriatal BDNF relationship. These findings suggest that STN DBS can drive BDNF in the parkinsonian brain and retains the potential for neuroprotection in PD.

## Introduction

The surgical approach of deep brain stimulation (DBS) of the subthalamic nucleus (STN) is effective in treating Parkinson's disease (PD) motor symptoms. STN DBS improves the quality of life of PD patients, in some cases to a greater extent than optimized pharmacotherapy (Deuschl et al., 2006; Martinez-Martin and Deuschl, 2007; Follett et al., 2010). Beyond its symptomatic efficacy, recent research has investigated whether STN DBS can also be disease-modifying. To date, clinical studies examining this issue have predominantly been retrospective studies of late-stage PD subjects who received DBS ≈12 years after diagnosis (Fischer and Sortwell, 2019). Results from these studies clearly indicate that when applied to late-stage PD, STN DBS is not neuroprotective. However, intervention in early-stage PD patients is likely required to completely answer this question. Evidence from preclinical studies in rats and monkeys have consistently demonstrated that unilateral or bilateral STN DBS pretreatment can prevent the degeneration of substantia nigra pars compacta (SNpc) dopamine (DA) neurons produced by DA-depleting neurotoxicants (Maesawa et al., 2004; Temel et al., 2006; Wallace et al., 2007). However, in contrast to results in neurotoxicant models, STN DBS applied in α-synuclein (α-syn) overexpression models has yielded mixed results with regards to neuroprotection (Fischer et al., 2017b; Musacchio et al., 2017). As a result, it remains uncertain whether STN DBS can protect the nigrostriatal system in the context of synucleinopathy.

PD models developed on the premise of elevated α-syn (i.e., overexpression) represented a step forward compared with their neurotoxicant model predecessors. However, whereas *SNCA*-linked familial PD is associated with moderately elevated α-syn, idiopathic PD is not characterized by elevated α-syn (for review, see Duffy et al., 2018a). In fact, the levels of α-syn achieved in viral vector-mediated overexpression models generally exceed those observed in idiopathic and even *SNCA* multiplication-linked PD (Volpicelli-Daley et al., 2016). Supraphysiological α-syn expression models may drive pathophysiological mechanisms not relevant to idiopathic PD. In the present study, we examine the impact of STN DBS on synucleinopathy in the α-syn preformed fibril (PFF) model, which triggers synucleinopathy in an environment of normal endogenous α-syn protein levels (Duffy et al., 2018a; Ugras et al., 2018). In this model, PFFs are internalized by neurons where they template and recruit endogenous α-syn into inclusions of insoluble phosphorylated α-syn (pSyn), analogous to Lewy bodies observed in idiopathic PD (Luk et al., 2009; Volpicelli-Daley et al., 2011, 2014; Zhou et al., 2011). The pSyn inclusions ultimately lead to neuronal dysfunction and degeneration. This toxicity is not because of the initial PFFs per se but can be directly tied to the recruitment of endogenous α-syn into inclusions; supported by the fact that PFFs do not induce toxicity when applied to α-syn^−/−^ neurons (Volpicelli-Daley et al., 2011; Luk et al., 2012).

In order to understand the neuroprotective potential of STN DBS, an understanding of the proposed neuroprotective mechanism is required. We have demonstrated that STN DBS increases brain-derived neurotrophic factor (BDNF) expression in the SN, the striatum, and the M1 cortex (Spieles-Engemann et al., 2011). Further, the STN-DBS associated neuroprotection appears dependent on this BDNF increase as neuroprotection is not observed when trkB signaling is prevented via an antagonist or α-syn overexpression (Fischer et al., 2017a,b). These results suggest that BDNF-trkB signaling is essential for protection of nigral neurons in the context of STN DBS. In the present study, we leverage our understanding of the time course and magnitude of synucleinopathy, inclusion-associated neuroinflammation, and nigral degeneration resulting from intrastriatal injection of α-syn PFFs in rats (Paumier et al., 2015; Duffy et al., 2018b; Polinski et al., 2018; Patterson et al., 2019a) to examine the neuroprotective impact of STN DBS. Further, we investigate the impact of α-syn inclusions themselves on levels of BDNF and nigrostriatal and corticostriatal BDNF relationships. Lastly, we evaluate the ability of STN DBS to drive BDNF production in the striatum and M1 cortex in the context of PFF-seeded synucleinopathy.

## Materials and Methods

### 

#### Experimental design and statistical analyses

**Figure 1.**
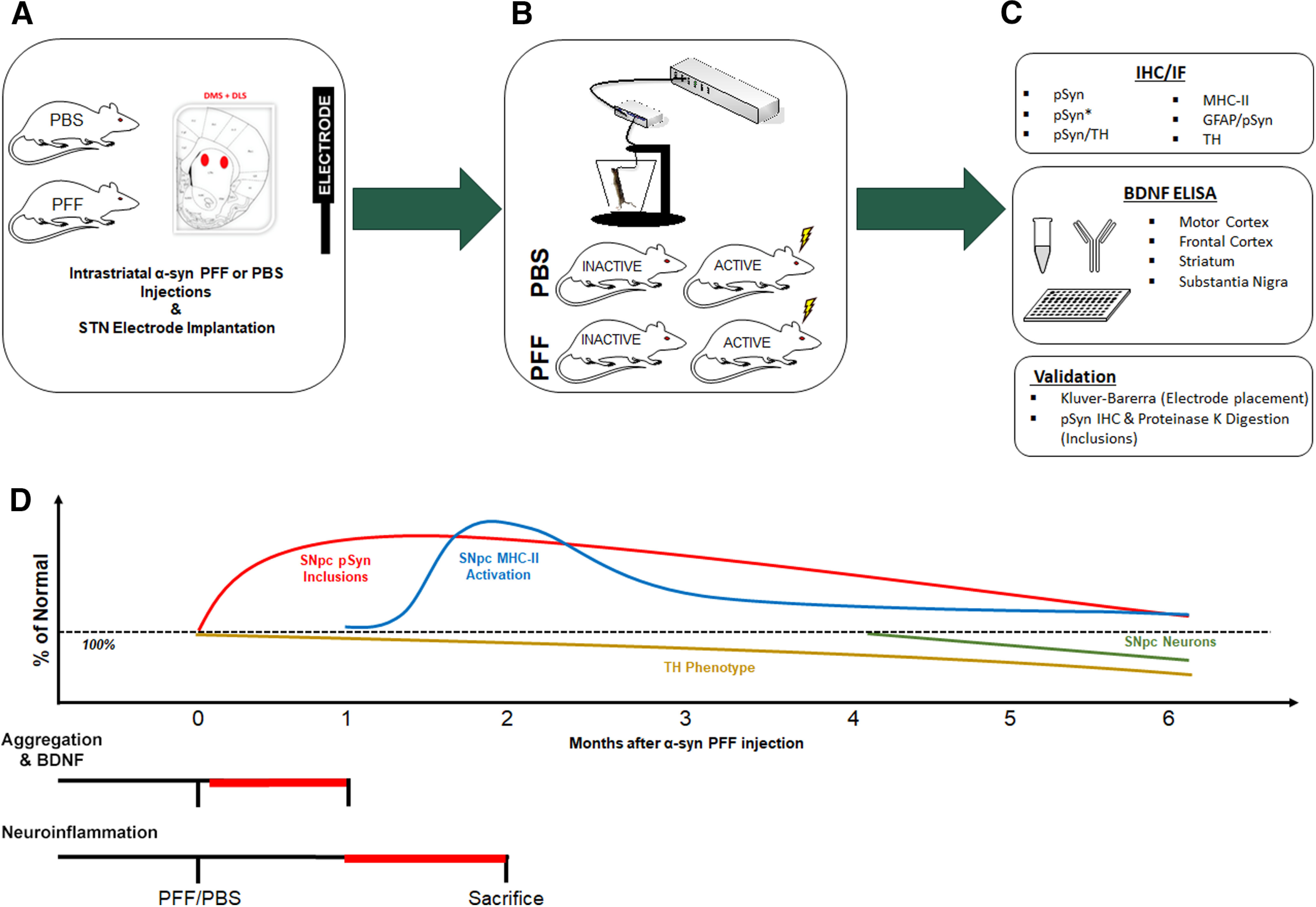
Experimental design. ***A***, Young (3 months old) male rats received two intrastriatal injections of α-syn PFFs or PBS and electrode implantation into the STN during the same surgical session. ***B***, Half of each treatment group was randomly assigned to receive continuous INACTIVE (stimulation never turned on) or ACTIVE stimulation. ***C***, Outcome measures include quantification of inclusions and ELISA probing for BDNF, quantification of MHC-IIir microglia, intensity, size and complexity of astrocytes, and validation of electrode placement (Kluver–Barerra) and validation of inclusion formation (proteinase K digestion). ***D***, Time points of stimulation interval and euthanasia were determined according to the period of peak inclusion formation (1–2 months, red), and microglial activation (2 months, blue) before overt loss of TH phenotype (2+ months, yellow) and degeneration of SNpc neurons (4–6 months, green). Red bars represent period of stimulation for each set of experiments.

All rats were unilaterally injected with saline (PBS) or preformed α-syn fibrils (PFF) into two sites in the dorsal striatum. During the same surgical session, all animals were implanted with an electrode targeting the STN. In the aggregation and BDNF cohorts, animals were randomly assigned 3 d after surgery to receive continuous stimulation (ACTIVE) or no stimulation (INACTIVE) for 30 d, corresponding with the beginning of the observed peak in aggregation (Duffy et al., 2018b; Patterson et al., 2019a). In the neuroinflammation cohort, animals were randomly assigned 30 d after surgery to receive ACTIVE or INACTIVE stimulation for 30 d, corresponding with the observed peak in activated microglia (Duffy et al., 2018b). An overview of the experimental design is presented in Figure 1.

Using previous PFF model data, new data for power calculations were simulated to represent the STN DBS intervention group from a Poisson distribution and then fit to a generalized linear model to determine whether differences could be detected. Results determined that only five rats per group would be required to detect a minimum of a 25% difference in pSyn aggregates because of DBS with over 90% power. Additional rats were then added per group based on our past experience with both the PFF and DBS models to account for injection failure rates, electrode failure over the month-long stimulation interval and improper electrode placement. Group sizes for each experiment are as follows: aggregation cohort, PFF INACTIVE (*n* = 15), PFF ACTIVE (*n* = 15); neuroinflammation cohort, PBS INACTIVE (*n* = 10), PBS ACTIVE (*n* = 6), PFF INACTIVE (*n* = 10), PFF ACTIVE (*n* = 9); BDNF cohort, PBS INACTIVE (*n* = 25), PBS ACTIVE (*n* = 5), PFF INACTIVE (*n* = 25), PFF ACTIVE (*n* = 15).

Statistical outliers were assessed using the Absolute Deviation from the Median method using the “very conservative” criterion (Leys, 2013). All statistical tests of the results were completed using GraphPad Prism software (version 6, GraphPad). All studies used two-way ANOVA, or independent samples *t* tests to assess differences between groups. The THir and soma fluorescence quantification and MHC-IIir quantification results were analyzed using two-way ANOVA with two treatment factors, stimulation and inclusions. The pSynir, pSyn*ir, and aggregation fluorescent quantification results were analyzed with two-tailed, independent samples *t* tests. Tukey's *post hoc* test was used on all ANOVA tests to determine significance between individual groups using the harmonic mean of the group sizes to account for unequal sample sizes. Linear regression modeling was used to model the relationship between sum pSyn intensity (predictor) and sum glial fibrillary acidic protein (GFAP) intensity (outcome) while including an interaction term between stimulation group and sum pSyn intensity. Total BDNF protein levels were analyzed using two-way ANOVA with two treatment factors: inclusions and hemisphere (to evaluate inclusion effects), or stimulation and hemisphere (to evaluate stimulation effects). Multivariate linear regression was used to model the stratified relationships between nigral BDNF, M1 cortical BDNF, frontal cortical BDNF, and striatal BDNF levels in rats by experimental group. For the analysis of combined frontal cortex, M1, and SN data, we used a mixed effects regression model with a random effect for sample ID to account for repeated measures. Striatal BDNF and experimental group were included as explanatory variables in all models. Linear models also included interaction terms between striatal BDNF (predictor) and experimental groups (categorical factor) to determine whether experimental treatment modified the relationship between M1/cortical/nigral and striatal BDNF. Control rats without PFF treatment or DBS stimulation were used as the reference group in all modeling. Statistical significance was set at α = 0.05.

#### Rats

Male, three-month-old Fischer 344 rats (Charles River) were given food and water *ad libitum* and housed in 12/12 h light/dark cycle conditions in the Grand Rapids Research Center, which is fully approved through the Association for Assessment and Accreditation of Laboratory Animal Care (AAALAC). All procedures were conducted in accordance with guidelines set by the Institutional Animal Care and Use Committee (IACUC) of Michigan State University.

#### Preparation and quality control of α-syn PFFs

**Figure 2.**
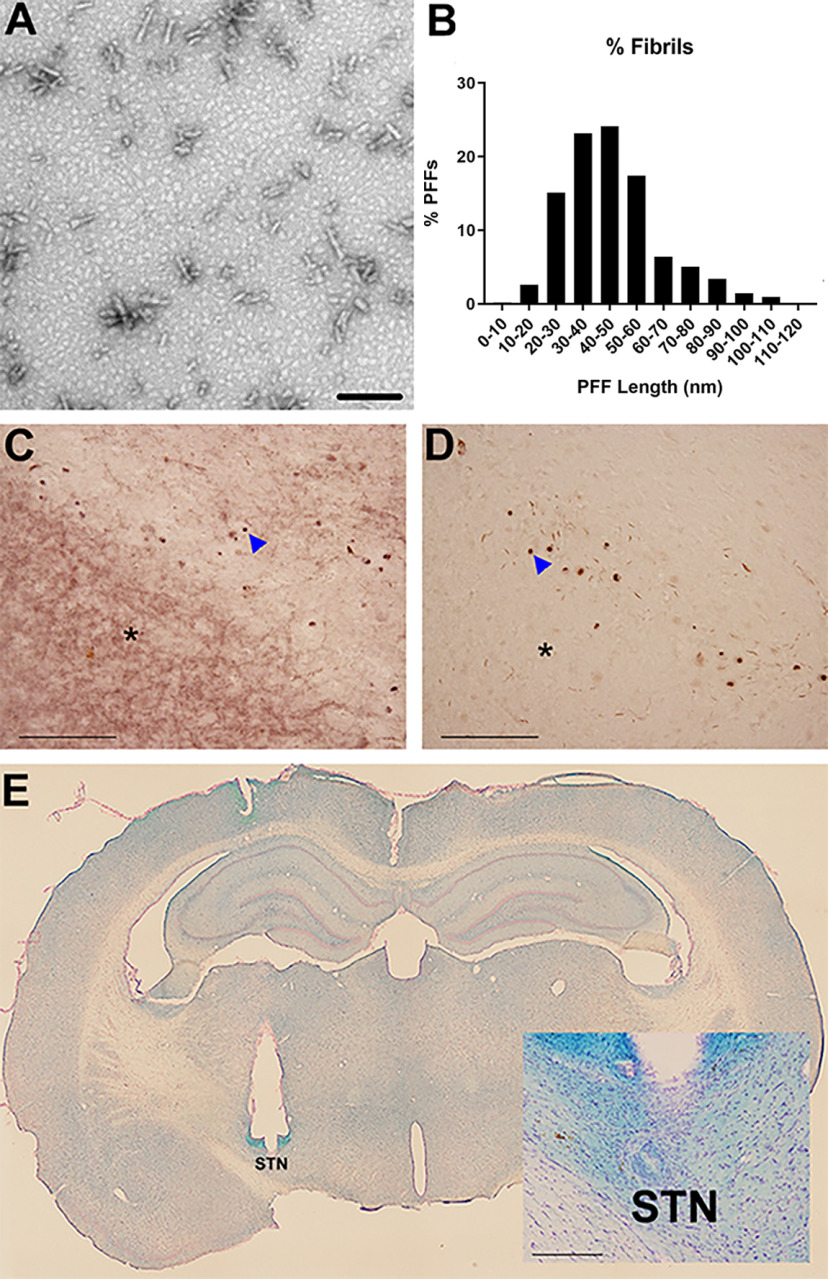
Validation of PFFs, inclusions, and electrode placement. ***A***, Fibrils were sonicated into small fragments. Scale bar: 100 µm. ***B***, The number of PFFs at each size is quantified revealing that the majority of sonicated fibrils were smaller than 60 nm. Nigral α-syn inclusions are proteinase K-resistant. Adjacent nigral sections were treated without (***C***) or with (***D***) proteinase K and stained for α-syn. Soluble α-syn in the substantia nigra pars reticulata (asterisk) is present without proteinase K, but is absent following treatment. Inclusions (arrow) remain after proteinase K treatment. ***E***, All rats included in the study were verified to have correct electrode placement in the STN. The location of the stimulating electrode before removal postmortem is evident. Inclusion criterion required the electrode tip to be within 250 µm of any part of the STN (as shown in inset, scale bar: 200 µm).

Recombinant, full-length mouse α-syn PFFs were prepared and verified as previously described (Volpicelli-Daley et al., 2011, 2014; Polinski et al., 2018). Before sonication, α-syn fibrils were assessed to verify lack of contamination (LAL assay; ∼1 endotoxin units/mg), are pelletable (sedimentation assay), cross-β-sheet conformation (thioflavin T), and structure (electron microscopy). Before injection, PFFs were thawed, diluted in sterile Dulbecco's PBS (DPBS; 4 μg/μl), and sonicated at room temperature using an ultrasonicating homogenizer (Q125 Sonicator; Qsonica) amplitude set at 30%, for 60, 1-s pulses, with 1 s between pulses (Patterson et al., 2019b). Following sonication, a sample of the PFFs was analyzed using transmission electron microscopy (TEM). Formvar/carbon-coated copper grids (EMSDIASUM, FCF300-Cu) were washed twice with ddH2O and floated for 1 min on a 10-μl drop of sonicated α-syn fibrils diluted 1:50 with DPBS. Grids were then stained for 1 min on a drop of 2% uranyl acetate aqueous solution; excess uranyl acetate was wicked away with filter paper and allowed to dry before imaging. Stained grids were imaged on a JEOL JEM-1400 TEM. The length of ∼500 fibrils per sample was measured to determine average fibril size (Fig. 2*A*,*B*).

#### Intrastriatal PFF injections

Intrastriatal α-syn PFF injections were conducted as described previously (Patterson et al., 2019a). Rats were anesthetized before surgery with isoflurane (5% induction, 1% maintenance). Each rat received two unilateral, intrastriatal injections (AP +1.0 mm, ML +2.0 mm, DV −4.0 mm and AP +0.1 mm, ML + 4.2 mm, DV −5.0 mm, AP and ML relative to bregma and DV relative to dura, injection rate 0.5 µl/min) of sonicated α-syn PFFs (total 16 µg in 2.0 µl/site). Sonicated PFFs were kept at room temperature during the duration of the surgical procedures. Injections were administered using a pulled glass needle attached to a 10-μl Hamilton syringe (Patterson et al., 2019b). After each injection, the needle was left in place for 1 min, retracted 0.5 mm, left in place for an additional 2 min, and then slowly withdrawn. Drill holes were filled with bone wax to prevent entry of dental cement during electrode placement.

#### Electrode implantation

Immediately following PFF injections, rats were unilaterally implanted (ipsilateral to α-syn PFF injections) with a bipolar, concentric microelectrode (inner electrode projection 1.0 mm, inner insulated electrode diameter 0.15 mm, outer electrode gauge 26, Plastics One) targeted to the dorsal border of the STN (AP 3.4 mm, ML 2.5 mm, relative to bregma, and DV 7.7 mm, relative to dura) as previously described (Spieles-Engemann et al., 2010a; Fischer et al., 2017b). The dorsal STN border placement site was selected to minimize damage to the nucleus, as has been described previously (Spieles-Engemann et al., 2010b). Burr holes were drilled in the skull; the electrode was fixed in place using bone screws, Metabond (Parkell), and dental acrylic. Animals were monitored daily postsurgery.

#### DBS

Three days following surgery (aggregation and BDNF cohorts) or 30 d following surgery (neuroinflammation cohort), rats were assigned to receive either stimulation (ACTIVE) for 30 d, or no stimulation for the same period (INACTIVE). Rats received STN stimulation that was continuously delivered in a freely moving setup as previously described (Spieles-Engemann et al., 2010b). Stimulation was generated by an Accupulser Signal Generator (World Precision Instruments) via a battery-powered Constant Current Bipolar Stimulus Isolator (World Precision Instruments). Stimulation parameters consisted of a frequency of 130 Hz, a pulse width of 60 µs, and an intensity of ∼50 µA. At the onset of stimulation, intensity settings were increased until orofacial or contralateral forepaw dyskinesias were observed, thereby confirming stimulation delivery. Immediately following a positive dyskinetic response, the intensity was set below the lower limit of dyskinesias (20–50 µA), such that no rat was functionally impaired by stimulation as previously described (Spieles-Engemann et al., 2010b).

#### Euthanasia

Rats were euthanized at the conclusion of each study (aggregation and BDNF cohorts, one month; neuroinflammation cohort, two months). Separate euthanasia methods were used for the two cohorts, as described below.

For the aggregation and BDNF cohorts, rats were deeply anesthetized (60 mg/kg, pentobarbital, i.p.) and perfused intracardially with heparinized normal saline at 37°C followed by ice-cold normal saline. Care was taken to minimize the tissue damage resulting from removing the skull with the electrode still intact. Brains were hemisected on the coronal plane at the optic chiasm. The caudal half was postfixed in 4% paraformaldehyde (PFA) for one week and transferred to 30% sucrose in 0.1 m phosphate buffer until sinking, and then used for the aggregation analyses. The rostral half was immediately flash-frozen in 2-methyl butane on dry ice and stored at 80°C until microdissected for BDNF analyses (M1 cortex and striatum). A subset of brains was immediately flash-frozen in 2-methyl butane on dry ice and stored at 80°C until microdissected for BDNF analyses (frontal cortex and substantia nigra).

For the neuroinflammation cohort, rats were deeply anesthetized (60 mg/kg, pentobarbital, i.p.) and perfused intracardially with heparinized normal saline at 37°C followed by 4% PFA. Care was taken to minimize the tissue damage resulting from removing the skull with the electrode still intact. Brains were postfixed in 4% PFA for 2 d and transferred to 30% sucrose in 0.1 m phosphate buffer until sinking and used for the neuroinflammation analyses.

#### Tissue processing for brightfield immunohistochemistry

Brains were frozen and sectioned on a sliding microtome at 40 μm. Free-floating sections (1:6 series) were transferred to 0.1 m tris-buffered saline (TBS). Following the washes, endogenous peroxidases were quenched in 3% H_2_O_2_ for 1 h and rinsed in TBS. Sections were blocked in 10% normal goat serum (NGS)/0.5% Triton X-100 (Tx-100) in TBS (NGS, Invitrogen; Tx-100, Fischer Scientific) for 1 h. Following the blocking, sections were immunolabeled with primary antibodies: pan rabbit-anti α-syn (Abcam; AB15530, 1:1000), mouse anti-pSyn at serine 129 (pSyn, 81A; Abcam; AB184674; 1:10,000), rabbit anti-pSyn truncated adamant and reactive (pSynSTAR, pSyn*; GeneTex; GTX50222; 1:2000), rabbit anti-tyrosine hydroxylase (TH; Millipore; MAB152, 1:4000), mouse anti-major histocompatibility complex-II for antigen-presenting microglia (MHC Class II RT1B clone OX-6, Bio-Rad; MCA46G, 1:5000), or mouse anti-GFAP (Millipore; AP124B, 1:2000) overnight in 1% NGS/0.5% Tx-100/TBS at 4°C. Following the washes, sections were incubated in biotinylated secondary antibodies followed by washes in TBS and 2-h incubation with Vector ABC standard detection kit (Vector Laboratories; PK-6100). Immunolabeling for pSyn, pSyn*, and TH was visualized by development in 0.5 mg/ml 3,3′ diaminobenzidine (DAB; Sigma-Aldrich; D5637-10G) and 0.03% H_2_O_2_. MHC-II was developed and visualized according to the manufacturer's instructions using the Vector ImmPACT DAB Peroxidase kit (Vector Labs; SK-4605). Slides were dehydrated in an ascending ethanol series and then cleared with xylenes before coverslipping with Cytoseal (Richard-Allan Scientific). pSyn-labeled sections were also counterstained with cresyl violet for quantification of intraneuronal pSyn inclusions in the SNpc.

#### Kluver–Barrera histology

Saline-perfused/PFA-postfixed brains (caudal half after hemisection) were frozen on dry ice and sectioned at 40-µm thickness using a sliding microtome in six series. Every sixth section of the STN was stained using Kluver–Barrera histochemistry (Kluver and Barrera, 1953) to evaluate for appropriate targeting of the electrode to the STN. Only rats with correctly positioned electrodes were included in the data analysis (Fig. 2*E*). Electrode location was considered to be appropriate if the tip of the electrode was observed within 250 µm of the border of the STN within any of the sections based on previous estimations of current spread conducted using similar stimulation parameters (Spieles-Engemann et al., 2010a).

#### Proteinase K digestion

To verify that PFF-induced α-syn inclusions were insoluble (Lewy body-like) as previously described (Li et al., 2010; Tanji et al., 2010), a subset of nigral sections was treated with or without 10 μg/ml proteinase K (Invitrogen; #25530015) then stained for pan rabbit-anti α-syn (Abcam; AB15530, 1:1000) as described above (Fig. 2*C*,*D*).

#### Microdissections of SNpc, M1 cortex, frontal cortex, and striatum

After brain removal, whole brains (neuroinflammation cohort) and rostral brains (aggregation and BDNF cohorts) were flash frozen in 2-methylbutane on dry ice and stored at −80°C. Microdissections were performed by thawing brains at −20°C for 30 min, then sectioning in a Leica 305S cryostat (Leica Biosystems) kept at −12°C; 2-mm^2^ punches were taken bilaterally from the M1 motor cortex and striatum, and unilaterally from the frontal cortex; 1-mm^2^ punches were taken bilaterally from the substantia nigra. During the procedure all brains were transferred on dry ice. Samples were stored at −80°C.

#### BDNF ELISA

Samples were thawed on ice and 250 µl of RIPA lysis buffer system was added to each sample (sc-249-48, Santa Cruz Biotechnology). Samples were sonicated, on ice, in short bursts (5–7 s) to homogenize, followed by a 30-min incubation on ice. A total of 10 µl was taken for BCA Assay (see below) and the remaining 240 µl was centrifuged at 10,000 × *g* for 30 min at 4°C and the supernatant collected. The ELISA was completed with duplicate samples, following the kit's protocol (BEK-2211, Biosensis).

#### BCA assay

A total of 10 µl of homogenized sample was diluted into 30 µl of 2% SDS. Following this step, samples were handled at room temperature. Samples were loaded in 10-µl triplicates into a 96-well plate and treated with 200 µl of prepared BCA reagent (50 reagent A:1 reagent B; 23250; ThermoFisher). Plates were incubated in the dark at 37°C for 30 min, then read on a Synergy H1 microplate reader (BioTek). Sample concentrations were calculated by comparing to a standard curve of known concentrations of bovine serum albumin.

#### Unbiased stereology of TH-immunoreactive (THir) and HuC neurons in the SNpc

The number of THir and HuC neurons in the SNpc ipsilateral and contralateral to α-syn PFF injection was estimated using unbiased stereology with the optical fractionator principle. Using a Nikon Eclipse 80i microscope, Retiga 4000R camera (QImaging) and Microbrightfield StereoInvestigator software (Microbrightfield Bioscience), neuron quantification was completed by drawing a contour around the SNpc borders using the 4× objective on every sixth (9 –11 sections per brain) and counting immunoreactive neurons according to stereological principles at 60× magnification. Briefly, counting frames (50 × 50 µm) were systematically and randomly distributed over a grid (183 × 112 µm) overlaid on the SNpc. A coefficient of error <0.10 was accepted. Data are reported as total estimates of THir or HuCir neurons in each hemisphere.

#### Total enumeration of pSyn and MHC-IIir cells

Because of heterogeneity in the distribution of both pSyn and MHC-IIir profiles within the SNpc, total enumeration rather than counting frames was used for quantification. Neurons with intraneuronal inclusions were defined as profiles of dark, densely stained immunoreactivity within cresyl violet-positive neurons. Contours were drawn around the SNpc using the 4× objective on every sixth section through the entire SNpc (9–11 sections). pSyn inclusion-containing neurons and MHC-IIir microglia were systematically counted within each contour using the 20× objective. Numbers represent the raw total number of pSyn inclusion-containing SNpc neurons or MHC-IIir microglia per animal multiplied by 6 to extrapolate the population estimate.

#### TH and pSyn double label immunofluorescence

Sections of rat tissue were blocked in 10% normal goat serum for 1 h and subsequently transferred to the primary antisera (TH: Millipore Ab152, rabbit anti-TH, 1:4000; and pSyn: Abcam AB184674, mouse anti-pSyn, 1:10,000) to incubate overnight at 4°C. Following primary incubation, tissue was incubated in the dark in secondary antisera against rabbit IgG (Invitrogen A11034, Alexa Fluor 488 goat anti-rabbit IgG, 1:500) and mouse IgG2a (Invitrogen A21135, Alexa Fluor 594 goat anti-mouse IgG2a, 1:500) for 1 h at room temperature. Sections were mounted on subbed slides and coverslipped with Vectashield Hardset Mounting Medium (Vector Laboratories H1400).

Images were taken on a Nikon 90i fluorescence microscope with a Nikon DS-Ri1 camera under identical exposure parameters. The eight median pSyn-seeded animals were included from each group: PFF-injected, active (5536 ± 180) or PFF-injected, inactive (5781 ± 223) with the three sections containing the most pSyn immunoreactive nigral neurons selected for analysis. Images were taken with the 20× objective so that the entire ipsilateral nigra was included with no overlap between images (7–16 frames per section). Outcome measure values from each animal were averaged and treated as sample replicates to form a single mean for each animal. Figures were produced in Photoshop 7.0. Brightness, saturation, and sharpness were adjusted only as necessary to best replicate the immunostaining as viewed directly under the microscope. Individual THir SNpc neurons were manually outlined using the NIS Elements Software Draw Bezier ROI tool (NIS Elements, Nikon Instruments). Only cells in the focal plane were analyzed. Within the outlined regions of interest (ROIs), pSyn intensity (mean intensity of ROI), TH intensity (mean intensity of ROI), and ROI area were evaluated. Intensity measures the average TH intensity of each pixel in the ROI. All pSynir aggregates were automatically outlined, and manually verified using the NIS Elements Software Auto Detect ROI tool (Nis Elements, Nikon Instruments). Only aggregates in the focal plane were analyzed. Aggregates were considered individual aggregates if there was a clear separation of space between neighboring inclusions (clumps that could not be delineated were treated as a single aggregate). Within outlined ROIs, pSyn intensity (mean pSyn intensity), and ROI area were evaluated.

#### GFAP and pSyn double label immunofluorescence

Sections of nigral rat tissue were blocked in 10% normal goat serum for 1 h and subsequently transferred to the primary antisera (GFAP: Millipore AP124B, mouse IgG1 anti-GFAP 1:2000; and pSyn: Abcam AB184674, mouse IgG2a anti-pSyn, 1:10,000) to incubate overnight at 4°C. Following primary incubation, tissue was incubated in the dark in secondary antisera against mouse IgG1 (Invitrogen A21121, Alexa Fluor 488 goat anti-mouse IgG1, 1:500) and mouse IgG2a (Invitrogen A21135, Alexa Fluor 594 goat anti-mouse IgG2a, 1:500) for 1 h at room temperature. Sections were mounted on subbed slides and coverslipped with Vectashield Hardset Mounting Medium (Vector Laboratories H1400).

Images were taken on a Nikon 90i fluorescence microscope with a Nikon DS-Ri1 camera. Figures were produced in Photoshop 7.0. Brightness, saturation, and sharpness were adjusted only as necessary to best replicate the immunostaining as viewed directly under the microscope. Three sections highly seeded with pSyn inclusions (or sections from PBS-injected controls corresponding to the same anterior-posterior coronal coordinates) were analyzed from each rat. Stitched images were taken with the 10× objective spanning the entire nigra. Outcome measure values from each animal were averaged and treated as sample replicates to form a single mean for each animal. To determine the immunofluorescence intensity of GFAP-labeled astrocytes, the SNpc was manually outlined based on TH immunoreactivity using the NIS Elements Software Draw Bezier ROI tool (NIS Elements, Nikon Instruments). Within the outlined ROI, GFAP intensity (mean immunofluorescence intensity of ROI), and pSyn immunofluorescence intensity (mean intensity of ROI) were measured and recorded in each appropriate channel.

#### Sholl analysis

Three high magnification (40×) z-stack images were taken from heavily seeded sections of nigra for each animal using NIS Elements software (NIS Elements, Nikon Instruments). Within each image, an astrocyte was selected and morphologic assessments were conducted as previously described (Tavares et al., 2017). Briefly, z-stacks were loaded into FIJI-ImageJ (Public Domain) and processes were reconstructed. Postreconstruction morphologic analyses included process length and number. Values represent the mean of the three replicates for each rat (total *n* = 30, 6–8 rats per treatment group). A 2D-rendered image was produced for each sample.

## Results

### Number and size of α-syn inclusions is not impacted by STN DBS

**Figure 3.**
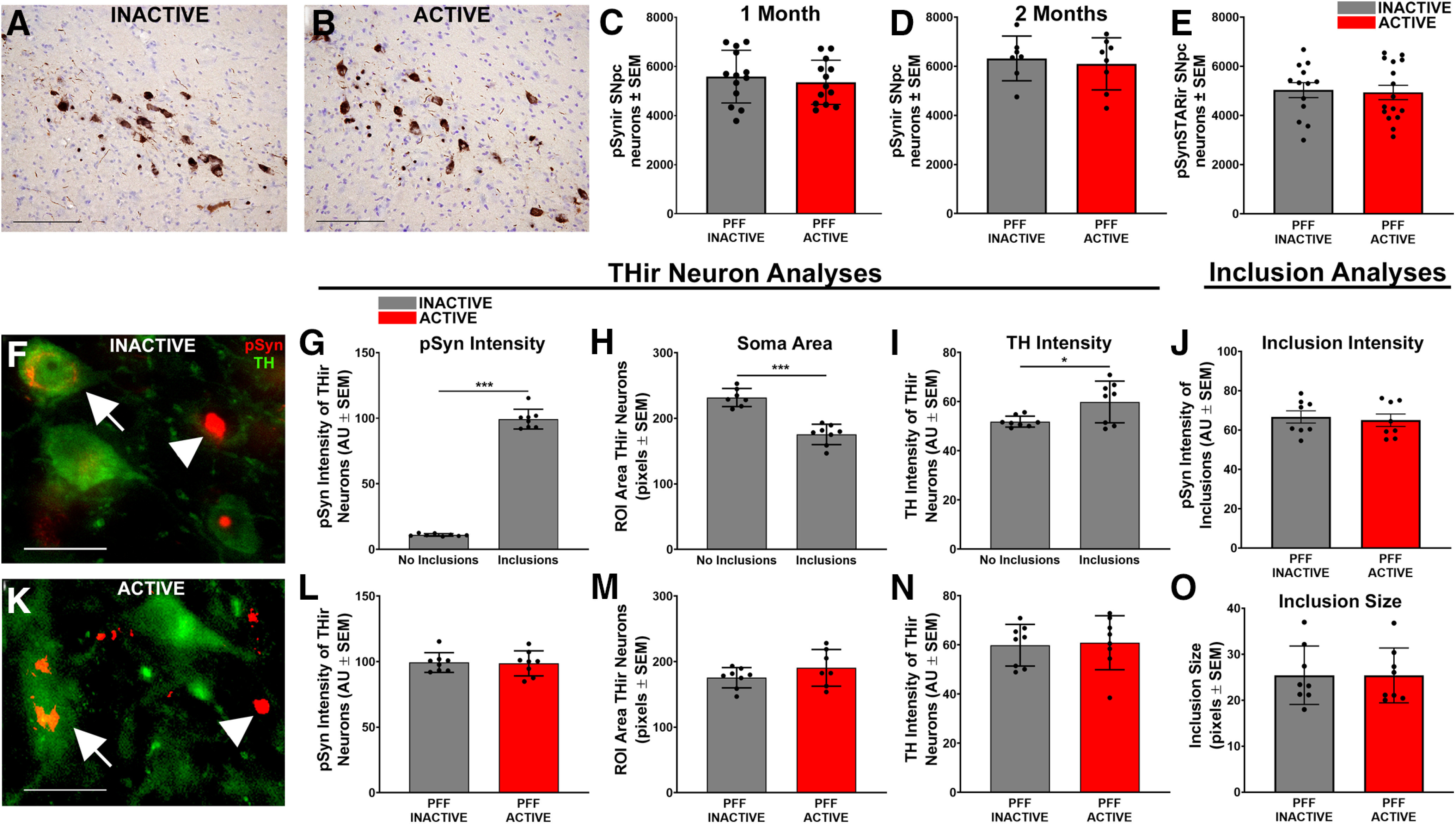
α-syn aggregates in the SNpc of rats are not impacted by STN DBS. There was no impact of STN DBS on the number of pSynir and pSynSTARir SNpc neurons in rats receiving INACTIVE stimulation (***A***) and ACTIVE stimulation (***B***). The number of pSynir neurons at one month (***C***), two months (***A*, *B*, *D***) and pSynSTARir neurons at one month **(E)** was not impacted by STN DBS. Scale bars: 100 µm. Immunofluorescence of THir neurons (green) and pSyn inclusions (red) were quantified (arrows in ***F***, ***K***). Examples of SNpc neurons with mature, compact pSyn inclusions that lack TH immunofluorescence, and thus were not included in analysis, are indicated by arrowheads. Scale bars: 25 µm. The effect inclusions have on mean pSyn intensity, soma area, and mean TH intensity of individual THir neurons within a PFF-treated rat are quantified ***G–I***, pSyn intensity increased from 10.97 ± 0.34 arbitrary units (AU) in neurons without inclusions to 99.34 ± 2.67 AU in neurons containing inclusions (***G***). Soma area decreased from 231.83 ± 5.20 pixels without inclusions to 175.45 ± 5.49 pixels with inclusions (***H***). TH intensity increased from 51.89 ± 0.79 AU in neurons without inclusions to 59.89 ± 2.30 AU in neurons with inclusions (***I***). STN DBS had no effect on pSyn intensity (ACTIVE = 98.68 ± 3.38 AU; ***L***), soma area (ACTIVE = 190.52 ± 10.58 pixels; ***M***), or TH intensity (ACTIVE = 60.90 ± 3.89 AU; ***N***). pSyn immunofluorescence intensity (***J***) and size (***O***) of individual pSynir inclusions were similar in INACTIVE STN DBS (***F***) and ACTIVE (***K***) STN DBS; *p ≤ 0.05, ***p ≤ 0.001. Values represent the mean ± SEM.

We examined the impact of STN DBS on α-syn inclusions in the SNpc following a month of stimulation during month 1 or month 2 following PFF injection. All rats injected with PFFs exhibited numerous inclusions immunoreactive for pSyn and pSyn* within neurons in the SNpc ipsilateral to α-syn PFF injection (Fig. 3*A*,*B*). At the one-month time point, we observed 5580 ± 297.2 pSyn-ir neurons in the ipsilateral SNpc of rats that received electrodes that were never activated (INACTIVE) compared with 5351 ± 249.1 pSyn-ir neurons in the ipsilateral SNpc of rats that received STN DBS (ACTIVE; Fig. 3*C*). At the two-month time point, we observed 6096 ± 375.0 pSyn-ir neurons in the ipsilateral SNpc of rats that received electrodes that were never activated (INACTIVE) compared with 6318 ± 343.8 pSyn-ir neurons in the ipsilateral SNpc of rats that received STN DBS (ACTIVE; Fig. 3*D*). STN DBS did not impact the number of pSyn-ir inclusion-bearing neurons in PFF-treated rats at either time point (one month: *p* = 0.5597; two months: *p* = 0.6931).

Autophagic degradation of pSyn results in a species of pSyn that is truncated at both terminals. This truncated form, termed pSyn* (pSyn-STAR; pSyn that is truncated, adamant and reactive), has been proposed to be the toxic species of pSyn (Grassi et al., 2018). To determine whether STN DBS impacted the formation of pSyn* the number of immunoreactive (pSyn*ir) nigral neurons were also quantified at the one-month time point. We observed no difference in the number of pSyn*ir nigral neurons in rats that received STN DBS (ACTIVE = 4931 ± 292.5) compared with those that did not (INACTIVE = 5028 ± 304.5; Fig. 3*E*, *p* = 0.8208).

### STN DBS does not alter pSyn-associated effects on nigral THir neurons

Qualitatively we observed two distinct populations of neurons within the SNpc possessing pSyn inclusions: (1) neurons with mature, condensed pSyn-ir inclusions with little or no TH immunofluorescence, presumably in later stages of inclusion formation (Fig. 3*F*,*K*); and (2) neurons with diffuse pSyn immunofluorescence, presumably in an earlier stage of inclusion formation, that colocalized for TH (Fig. 3*F*,*K*). In this second category of neurons with identifiable TH immunofluorescence we examined the impact of pSyn inclusions on average TH immunofluorescence intensity, and soma size. Not surprisingly, THir SNpc neurons with inclusions exhibited a 9× increase in pSyn intensity (*p* < 0.0001) compared with neighboring THir neurons without inclusions (Fig. 3*G*). Further, THir SNpc neurons with pSyn inclusions displayed roughly 25% decreased soma size (Fig. 3*H*, *p* < 0.0001), and 13% increased TH intensity (Fig. 3*I*, *p* = 0.042). We next examined whether STN DBS stimulation status impacted inclusion-associated alterations within nigral THir neurons. STN DBS had no effect on pSyn intensity, TH soma size, or TH intensity (Fig. 3*L–N*, *p* = 0.9606, *p* = 0.9864, and *p* = 0.3688, respectively) of THir nigral neurons bearing pSyn inclusions. These results suggest that the early formation of pSyn inclusions within nigral DA neurons alters TH expression and compromises neuronal size but that these inclusion-associated effects are not impacted by STN DBS. This also suggests that STN DBS does not induce the degradation of α-syn inclusions following formation.

We also tested whether the size and immunofluorescence intensity of individual pSyn immunoreactive aggregates at the one-month time point were impacted by STN DBS (Fig. 3*J*,*O*). Neither the size nor pSyn intensity of pSyn aggregates were affected by stimulation (*p* = 0.9914 and *p* = 0.7090, respectively). Together, these results suggest that STN DBS during the initial months of inclusion formation does not impact the accumulation or degradation of α-syn inclusions in the SNpc.

### STN DBS does not impact early loss of SNpc phenotype

**Figure 4.**
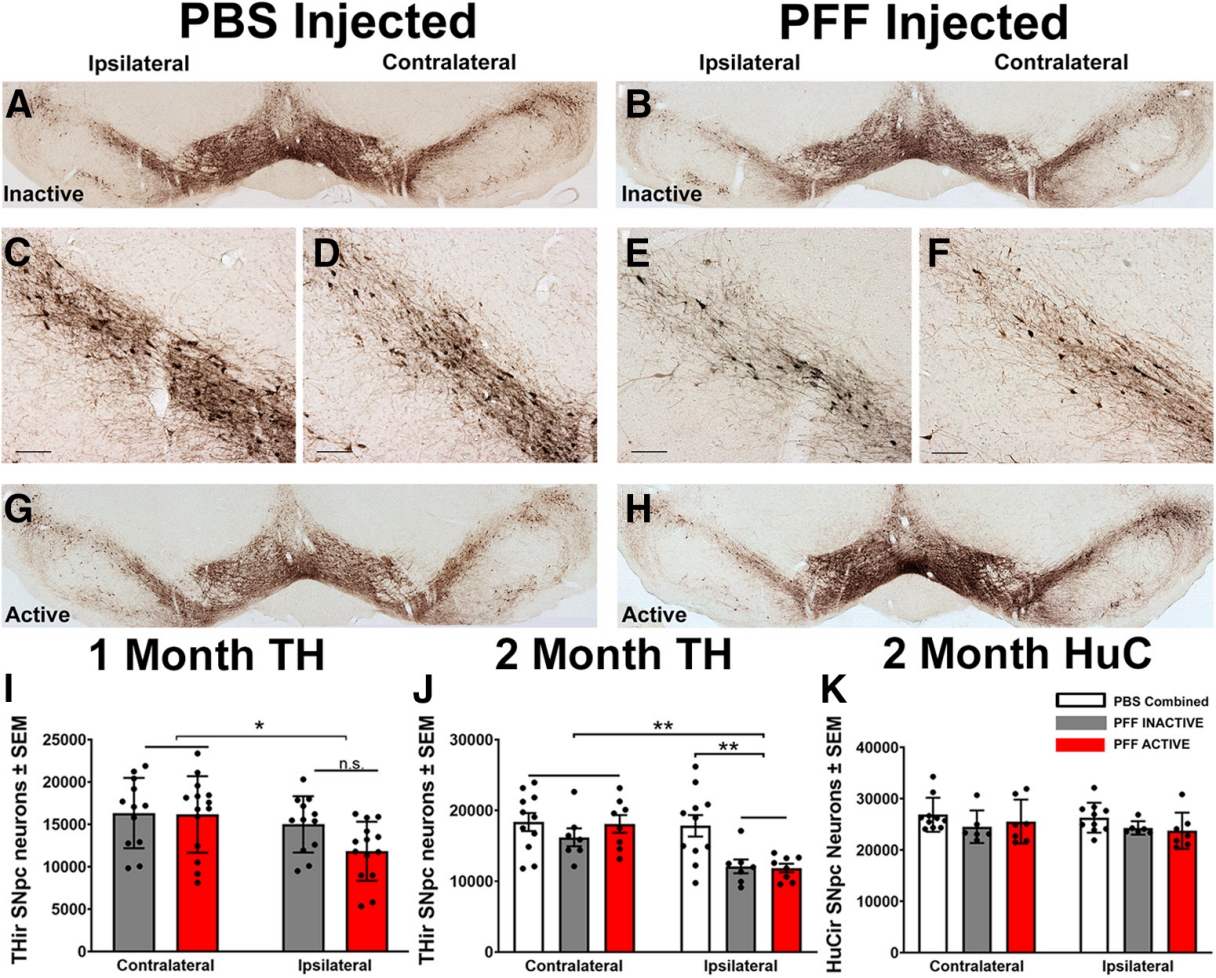
STN DBS does not impact PFF-inclusion associated loss of TH phenotype in nigral neurons in PFF-treated rats one or two months after injection. Whole nigra stiched images ***A***, ***B***, ***G***, ***H*** and high-magnification ipsilateral boxed insets **(*C***, ***E***, ***D***, ***F***, respectively) of THir neurons two months after PFF treatment. Quantification of nigral THir neurons in rats without (INACTIVE) and with (ACTIVE) STN DBS one month (***I***) and two months (***J***) after PFF treatment demonstrate an inclusion-associated decrease that is not affected by stimulation status. INACTIVE (***A***, ***C***) and ACTIVE (***G***, ***D***) PBS-treated rats were statistically similar and combined (***J***). Contralateral THir counts were not different across groups. Ipsilateral THir counts were decreased in both INACTIVE (***B***, ***E***) and ACTIVE (***H***, ***F***) PFF-treated rats compared with their contralateral side or PBS-treated controls (***J***). Loss of TH phenotype in the SN was not associated with neurodegeneration as measured by HuC (pan neuronal marker; ***K***). Scale bars: 100 µm; *p ≤ 0.05, **p ≤ 0.01. Values represent the mean ± SEM.

Previous studies using identical intrastriatal α-syn PFF injection parameters revealed significant decreases in ipsilateral SNpc THir neurons at four and six, but not two, months after injection (Patterson et al., 2019a). In the present study, we observed a modest, yet significant decrease (∼18%) in THir neurons in the SNpc ipsilateral to PFF injection at one month (*p* = 0.012; Fig. 4*I*) and a greater loss of THir neurons (∼33%) at two months (*p* = 0.006; Fig. 4*A–H*,*J*). However, when comparing THir neurons in the ipsilateral inclusion-bearing hemispheres of rats that received STN DBS (one-month ACTIVE: 11,842 ± 931.2; two-month ACTIVE: 11,878 ± 602.4) versus THir neurons in rats that received no stimulation (one-month INACTIVE: 15,004 ± 959.9; two-month INACTIVE: 12,099 ± 972.2), we observed no significant differences (one month: *p* = 0.1321, two months: *p* > 0.9999; Fig. 4*I*,*J*). These results suggest that STN DBS does not impact the ipsilateral decrease of THir SNpc neurons induced by α-syn PFF injection. To determine whether the loss of TH phenotype observed reflected loss of TH phenotype or neurodegeneration, we quantified the total number of SNpc neurons using the pan-neuronal marker, HuC. We observed no difference in the number of nigral neurons as a result of PFF injections or stimulation (Fig. 4*K*). Specifically, HuC-ir neurons in PFF-injected rats that received STN DBS (ipsilateral: 23,765 ± 1331.4; contralateral: 25,564 ± 1612.5) and rats that received no stimulation (ipsilateral: 24,306 ± 535.0; contralateral: 24,533 ± 1296.6) showed no significant differences from PBS-injected rats (combined ipsilateral: 26,303 ± 922.8; combined contralateral: 26,882 ± 1047.6; *p* = 0.9996). Collectively, these data suggest that loss of THir SNpc neurons observed ipsilateral to PFF injection reflects loss of TH phenotype but not neuronal degeneration.

### STN DBS does not impact α-syn inclusion-triggered microgliosis and astrogliosis

**Figure 5.**
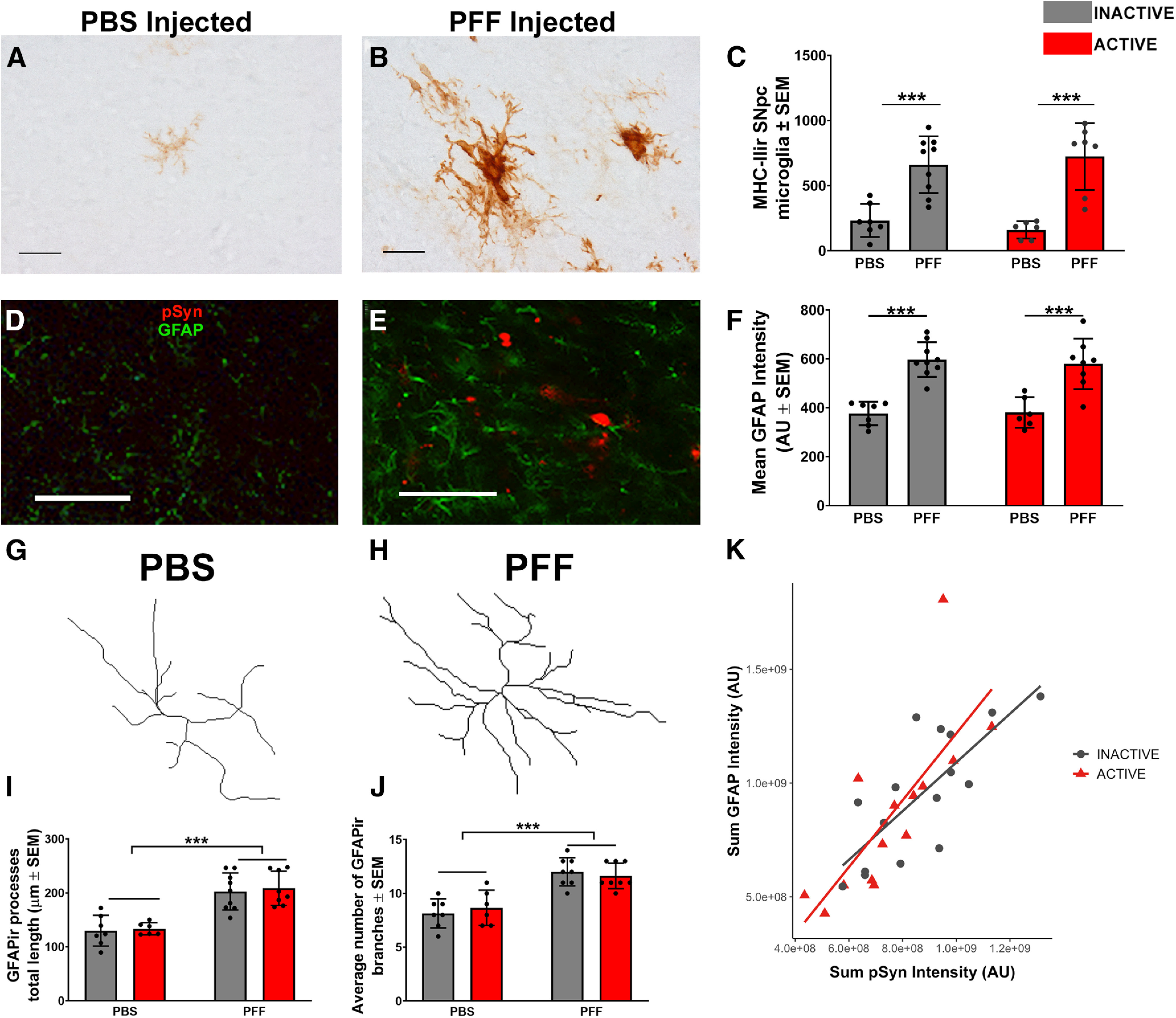
α-Syn inclusion-associated microgliosis and astrogliosis in the SN is not impacted by STN DBS. α-Syn inclusions induced by PFF injection are associated with significantly more MHC-IIir microglia in the SN at the two-month time point compared with rats that received control intrastriatal PBS injections in which inclusions do not form (***A***, ***B***). STN DBS did not impact the number of MHC-IIir microglia (***C***). Scale bars: 100 µm. pSyn (red) and GFAP (green) fluorescence intensity were measured in the SNpc of PBS-injected (***D***) and PFF-injected (***E***) rats. Mean GFAP intensity in PFF-treated rats is higher (INACTIVE = 597.7 ± 23.7 AU, ACTIVE = 580.0 ± 36.5 AU) than in PBS-treated controls (INACTIVE = 376.7 ± 18.2 AU, ACTIVE = 381.0 ± 25.6 AU, *p* < 0.0001; ***F***). Scale bars: 50 µm. GFAPir astrocytes from PBS-injected (***G***) and PFF-injected (***H***) rat nigras were outlined to produce 2D-rendered paths. Total length of all paths (***I***) and number of branches (***J***) were quantified as an indicator of astrocyte complexity. There is a significant positive association between total intensity of GFAP in arbitrary units and total intensity of pSyn (***K***); ***p ≤ 0.001. Values represent the mean ± SEM.

Formation of pSyn inclusions in the SNpc is associated with an increase in the number and size of MHC-IIir microglia two months following intrastriatal PFF injection (Duffy et al., 2018b). In the present experiment, we observed a similar pSyn-associated increase in MHC-IIir microglia at two months in the SN in rats injected with α-syn PFFs (693.1 ± 31.1) compared with control rats that received intrastriatal PBS injections (197.1 ± 36.1; Fig. 5*A–C*). Stimulation of the STN during month two did not impact the number of MHC-IIir microglia in the SN after PFF treatment (*p* = 0.9876).

Rats seeded to form pSyn inclusions in the SNpc displayed a ∼36% increase in GFAP immunofluorescence intensity at two months compared with PBS control rats at the same time point (Fig. 5*D–F*, *p* < 0.0001). STN stimulation did not impact GFAP immunofluorescence in either PBS or PFF-injected rats (*p* = 0.9996 and *p* = 0.9626, respectively). Consistent with our previous finding that ∼ 33% of nigral neurons contain inclusions at this time point (Patterson et al., 2019a), we observed a ∼33% increase in nigral pSyn intensity (data not shown). We further characterized the length and complexity of GFAPir astrocytic processes in the presence of pSyn inclusions and the impact of STN DBS using Sholl analysis (Fig. 5*G–J*). Astrocytes in PFF-injected rats possessing pSyn inclusions in SNpc neurons exhibited ∼36% longer total process length compared with those astrocytes in rats treated with PBS (*p* < 0.0001; Fig. 5*I*). Similarly, the number of astrocytic branches was ∼31% greater in inclusion-bearing rats compared with controls (*p* < 0.0001; Fig. 5*J*). STN DBS did not impact astrocytic process length or complexity in either PBS-treated or PFF-treated rats (Fig. 5*I*,*J*, *p* = 0.9964 and *p* = 0.9743, respectively). Finally, to determine the relationship between pSyn inclusions and GFAP expression in the SN, we used linear regression to model the relationship between the sum pSyn intensity and the sum GFAP intensity in PFF-treated rats, revealing a significant (β = 1.21, *p* < 0.0001) positive association (Fig. 5*K*). There was no significant difference in pSyn intensity between active and inactive stimulation (*p* = 0.9403), indicating that the relationship between pSyn and GFAP intensity was not altered by STN DBS treatment (Fig. 5*K*). Collectively, these experiments show that the accumulation of pSyn inclusions is associated with increased microglial and astroglial reactivity in the SN, and that stimulation of the STN does not alter these neuroinflammatory parameters.

### α-Syn inclusions do not change BDNF protein levels

**Figure 6.**
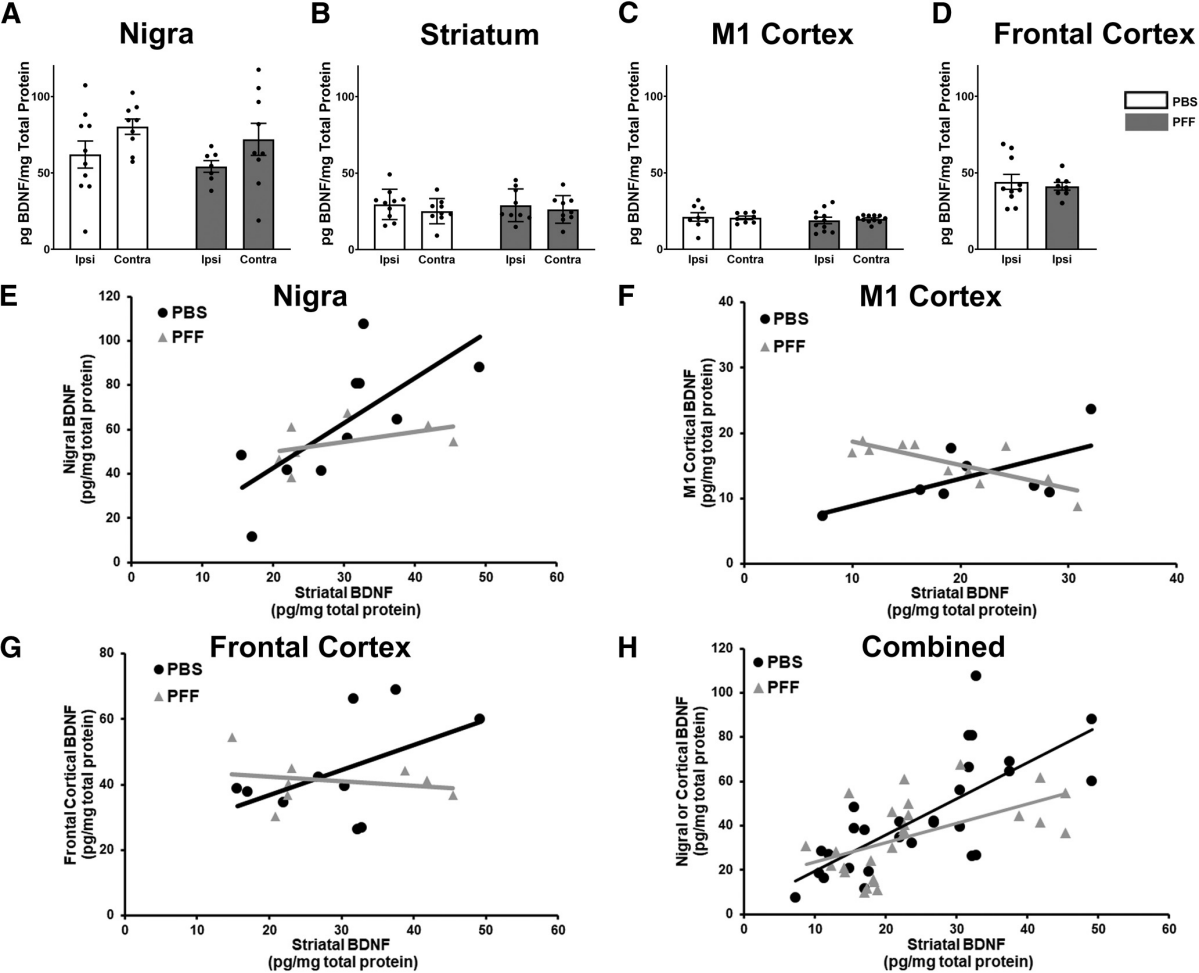
α-Syn inclusions alter nigrostriatal and corticostriatal BDNF relationships. Total BDNF protein levels measured in the SN (***A***), striatum (***B***), M1 cortex (***C***), or frontal cortex (***D***) ipsilateral to injection revealed no differences (*p* > 0.05) because of pSyn inclusions. ***E***, PBS-injected rats display a significant positive association between striatal and nigral BDNF protein levels (β = 2.02, SE = 0.58, *p* = 0.004) that is disrupted by treatment with PFFs, as reflected by the negative interaction term, however, is not significant (β = −0.90, SE = 0.56, *p* = 0.13). ***F***, In the M1 cortex, PBS rats show a significant positive association with striatal BDNF (β = 0.98, SE = 0.40, *p* = 0.03) that is significantly disrupted by PFFs (β = −2.67, SE = 0.68, *p* = 0.001). ***G***, The relationship between frontal cortical and striatal BDNF protein is positive, but not significant, in PBS-treated rats (β = 0.76, SE = 0.40, *p* = 0.08), and is disrupted, but not significant in PFF-treated rats (β = −1.58, SE = 0.91, *p* = 0.11). ***H***, When all structures are combined, there is a significant positive association between source (nigra/cortex) and striatal BDNF protein levels (β = 1.08, SE = 0.26, *p* = 0.0001) that is significantly disrupted by PFF treatment (β = −0.835, SE = 0.33, *p* = 0.02). Values represent the mean ± SEM.

PFF injection results in widespread accumulation of pSyn inclusions in neurons that innervate the striatum whose soma are located in the SNpc and multiple cortical areas, as well as pSyn immunoreactive neurites in the striatum. We first examined whether the accumulation of pSyn inclusions impacted baseline levels of BDNF in the absence of stimulation. Measurement of BDNF protein levels in the SN, striatum, M1 cortex, and frontal cortex revealed no differences in rats treated with PFFs versus PBS (*p* = 0.9083, *p* = 0.9991, *p* = 0.8175, and *p* = 0.6305, respectively; Fig. 6*A–D*). Furthermore, there were no differences in BDNF levels in the injected side compared with the contralateral side for any of the examined structures (SN: *p* = 0.3272, striatum: *p* = 0.9282, M1 cortex: *p* = 0.9136). Specifically, in the SN, BDNF levels (pg/mg protein) in PBS-injected rats were 62.05 ± 8.86 (ipsilateral) and 80.28 ± 5.01 (contralateral), compared with 54.26 ± 3.83 (ipsilateral) and 72.00 ± 10.47 (contralateral) in PFF-injected rats (Fig. 6*A*). In the striatum, BDNF levels in PBS-injected rats were 29.54 ± 3.14 (ipsilateral) and 25.09 ± 2.75 (contralateral), compared with 28.95 ± 3.57 (ipsilateral) and 26.25 ± 3.01 (contralateral) in PFF-injected rats (Fig. 6*B*). In the M1 cortex BDNF levels in PBS-injected rats were 21.13 ± 2.77 (ipsilateral) and 20.56 ± 1.13 (contralateral), compared with 18.95 ± 2.11 (ipsilateral) and 19.85 ± 0.71 (contralateral) in PFF-injected rats (Fig. 6*C*). Finally, in ipsilateral frontal cortex, BDNF levels in PBS-injected rats were 44.10 ± 4.90, compared with 41.18 ± 7.21 in PFF-injected rats (contralateral levels were not evaluated as they were used to verify α-syn inclusions after PFF injection; Fig. 6*D*).

### α-Syn inclusions alter nigrostriatal and corticostriatal BDNF relationships

The majority of striatal BDNF is produced and anterogradely transported from the cortex, with a smaller amount of striatal BDNF supplied from the substantia nigra (Altar et al., 1997; Conner et al., 1997). We therefore examined the relationship between BDNF levels in the cortex or SN and the striatum by comparing BDNF levels within individual rats treated with PBS or PFF (Fig. 6*E–H*). In PBS rats, there was a significant positive association between BDNF levels in the nigra and striatum (β = 2.02, SE = 0.58, *p* = 0.004; Fig. 6*E*). There was also a significant positive association between BDNF levels in M1 cortex and the striatum (β = 0.98, SE = 0.40, *p* = 0.03; Fig. 6*F*). Meanwhile, the association between BDNF levels in the frontal cortex and striatum was positive, but only approached significance (β = 0.76, SE = 0.40, *p* = 0.08; Fig. 6*G*). Of particular interest, in rats with PFF-seeded pSyn inclusions, all three of these associations between BDNF levels were significantly disrupted, as reflected by negative interaction terms between striatal BDNF and inclusion status in the three regression models: nigra (β = −0.90, SE = 0.56, *p* = 0.13), M1 cortex (β = −2.67, SE = 0.68, *p* = 0.001), and frontal cortex (β = −1.58, SE = 0.91, *p* = 0.11). While all three interactions between striatal BDNF and inclusion status were negative, only the individual M1 cortex interaction was significant. We used a mixed effects regression model to evaluate the overall effect of PFF compared with PBS (Fig. 6*H*). There was a significant positive association between BDNF levels in the striatum and BDNF in the combined nigra, M1 cortex, and frontal cortex (β = 1.08, SE = 0.26, *p* = 0.0001). In rats with PFF-seeded pSyn inclusions, this association between BDNF levels was significantly disrupted, as reflected by a negative interaction term between Striatal BDNF and inclusion status in the mixed effect model (β = −0.835, SE = 0.33, *p* = 0.02). These results suggest that pSyn inclusions disrupts normal corticostriatal and nigrostriatal BDNF relationships.

### STN DBS increases striatal BDNF

**Figure 7.**
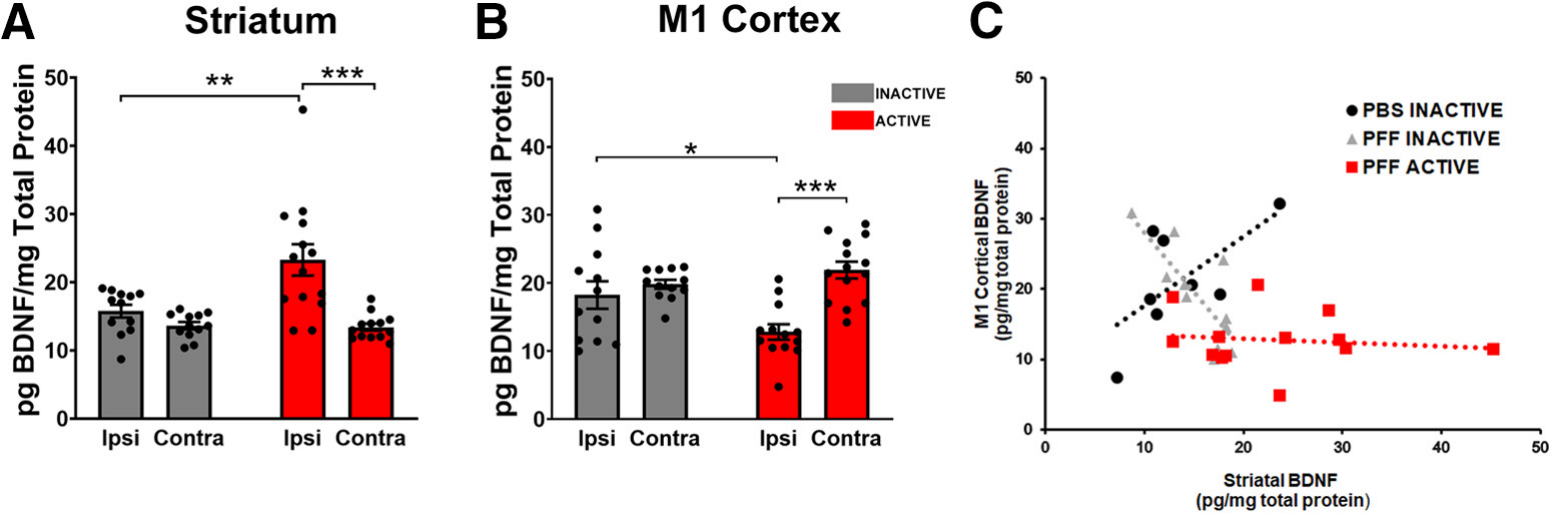
STN DBS increases striatal BDNF protein and partially reverses the inclusion-associated negative corticostriatal BDNF relationship. The relationship between cortical and striatal BDNF in ACTIVE STN DBS rats with α-syn inclusions increases ipsilateral BDNF protein in the striatum (25.74 ± 2.16 pg/mg) compared with INACTIVE (15.77 ± 0.94 pg/mg, *p* < 0.0001; ***A***). In contrast, BDNF protein in the ipsilateral M1 cortex (12.68 ± 1.06 pg/mg) of inclusion-bearing rats is decreased compared with INACTIVE rats (18.26 ± 2.03 pg/mg, *p* = 0.013; ***B***). Neither the contralateral striatum (13.42 ± 0.52 pg/mg) nor M1 (21.92 ± 1.23 pg/mg) show changes in BDNF protein following ACTIVE STN DBS (***A***, ***B***). The relationship between BDNF protein levels in M1 cortex and striatum is altered by PFFs and STN DBS (***C***). Control (PBS, INACTIVE) rats show a positive association between M1 cortical and striatal BDNF expression (black circles; β = 14.862, *p* = 0.006). This relationship is reversed in PFF-treated animals, resulting in a negative interaction between striatal BDNF and experimental group (gray triangles; interaction term β = −38.035, *p* < 0.0001). STN DBS in inclusion-bearing neurons partially mitigates this reversal caused by inclusions, as reflected in the decreased magnitude of the negative interaction term (red squares; interaction term β = −16.820, *p* < 0.05); *p ≤ 0.05, **p ≤ 0.01, ***p ≤ 0.001. Values represent the mean ± SEM.

We next evaluated how one month of continuous STN DBS impacts BDNF protein levels in the striatum and M1 cortex of rats with widespread accumulation of pSyn inclusions. STN DBS resulted in a ∼150% increase in BDNF protein in the ipsilateral striatum compared with the ipsilateral INACTIVE striatum (*p* = 0.002; Fig. 7*A*). However, ipsilateral BDNF levels in the M1 cortex of α-syn inclusion bearing rats were significantly decreased ∼30% compared with the ipsilateral INACTIVE M1 by STN DBS (*p* = 0.033; Fig. 7*B*). Interestingly, contralateral striatal and M1 cortical BDNF protein levels were unchanged by STN DBS (*p* = 0.9995 and *p* = 0.6826, respectively; Fig. 7*A*,*B*).

### STN DBS partially mitigates the corticostriatal BDNF association disrupted by α-syn inclusions

In control rats we observe a significant positive association between BDNF levels in either the frontal cortex, M1 cortex, or SN and the striatum (Fig. 6*E–G*). In a pilot cohort of naive rats treated with STN DBS for 5 d, this positive relationship remained (data not shown). We next evaluated the relationship between M1 BDNF levels and striatal BDNF levels with and without stimulation in PBS-treated and PFF-treated rats. INACTIVE PBS rats displayed a significant, positive association between M1 cortical and striatal BDNF protein levels (Fig. 7*C*, black circles, β = 14.862, *p* = 0.006). As we observed previously, the presence of pSyn inclusions in INACTIVE PFF rats was associated with a reversal of this relationship, resulting in a negative interaction term between striatal BDNF and experimental group (Fig. 7*C*, gray triangles; interaction term β = −38.035, *p* < 0.0001). However, STN DBS in PFF-treated rats partially mitigated this reversal associated with inclusions, as reflected in the decreased magnitude of the negative interaction term (Fig. 7*C*, red squares, interaction term β = −16.820, *p* = 0.011). Collectively, these data show that accumulation of pSyn inclusions has a negative impact on the relationship in BDNF levels between structures that innervate the striatum and the striatal target. Despite this consequence of pSyn inclusions, STN DBS increases total BDNF levels in the striatum and partially mitigates the disrupted corticostriatal BDNF relationship.

## Discussion

In the present study, we examined the impact of STN DBS during the early inclusion accumulation and neuroinflammation phases of the rat α-syn PFF model. We observed that continuous STN DBS does not impact the accumulation, size or intensity of pSyn inclusions in SNpc neurons. STN DBS has previously been associated with increased odds of higher α-syn pathology in the SN of PD subjects (Pal et al., 2017) however it was not concluded that STN DBS drives α-syn pathology because of limited sample size and the significantly longer disease duration of the DBS subjects (six years). While our present findings are difficult to compare to this study, they do not appear to be contradictory. Indeed, the artificially accelerated formation of pSyn inclusions triggered by α-syn PFF exposure may preclude our ability to examine the potential of STN DBS to impact the protracted accumulation of synuclein pathology characteristic of the disease state. In addition, STN DBS did not impact the microgliosis or astrogliosis associated with the formation of pSyn inclusions in SNpc neurons. Further, the formation of pSyn inclusions did not impact total levels of BDNF in structures in which pSyn inclusions accumulate: SNpc, M1 cortex, frontal cortex. However, the normally positive association between nigrostriatal and corticostriatal BDNF levels was negatively impacted in inclusion-bearing rats. Despite this, STN DBS rats significantly increased BDNF protein in the striatum of rats bearing pSyn inclusions, which partially restored the normal corticostriatal BDNF relationship.

We have consistently observed that intrastriatal α-syn PFF injections result in a predictable cascade of degenerative events in the nigrostriatal system that can be summarized as two distinct phases (Paumier et al., 2015; Duffy et al., 2018b; Patterson et al., 2019a). The earliest months following injection (months 1–3) are characterized by peak magnitude of accumulation of pSyn inclusions, peak microglial MHC-II expression and the initiation of TH phenotype loss in nigral soma in the absence of neuronal death. In subsequent months (months 4–6) pSyn inclusions decrease (presumably because of neuronal loss; Osterberg et al., 2015), loss of THir innervation in the striatum is observed followed by overt neurodegeneration of nigral soma with maximum degeneration detectable at approximately six months after injection. In our present experimental paradigm, we examined STN DBS effects during months 1–2 on pSyn aggregation, neuroinflammation, TH soma phenotype, and BDNF. Importantly, we sought to understand the impact of stimulation during the period of peak synucleinopathy. We demonstrate that during the time of peak α-syn accumulation STN DBS maintains the ability to elevate striatal BDNF levels and thereby partially restores the corticostriatal BDNF relationship that had been disrupted by the presence of pSyn inclusions. These results are consistent with our previous work demonstrating that STN DBS drives an increase in BDNF protein in the striatum of naive rats (Spieles-Engemann et al., 2011).

Our previous research also has shown that BDNF-trkB signaling in SNpc neurons is required for the neuroprotective effect of STN DBS in a neurotoxicant model (Fischer et al., 2017a). In the present study, we did not examine, nor could we examine based on the timing of stimulation, whether STN DBS prevents loss of striatal dopaminergic innervation, reverses loss of nigral TH phenotype or prevents the eventual degeneration of nigral neurons. As such, the present study cannot and does not provide evidence that DBS is associated with neuroprotection against α-syn inclusion-induced toxicity. However, the observation that STN DBS increases striatal BDNF despite the presence of pSyn inclusions suggests a mechanism that could be harnessed to exert neuroprotective effects on the later events of loss of dopaminergic terminals in the striatum and ultimate degeneration of nigral soma. Similar to PD (O'Malley, 2010; Burke and O'Malley, 2013; Kordower et al., 2013; Nordströma et al., 2015; Tagliaferro and Burke, 2016), dysfunction in nigrostriatal terminals precedes neuronal loss in the PFF model (Patterson et al., 2019a). An increase in striatal BDNF may offset this dysfunction via many possible mechanisms (Fischer and Sortwell, 2019). BDNF signaling increases DA release, TH synthesis, DA turnover, and DA neuron activity, all of which are decreased in PD (Knusel et al., 1991; Beck et al., 1993; Altar et al., 1994; Hyman et al., 1994; Shen et al., 1994; Zhou et al., 1994; Blöchl and Sirrenberg, 1996; Benskey et al., 2016). This is supported by preclinical evidence that STN DBS alters dopaminergic neurotransmission (Stefani et al., 2017), perhaps contributed to by increased BDNF signaling. Further, in the striatum BDNF helps maintain postsynaptic spine density (Rauskolb et al., 2010), which is decreased in postmortem PD brains (McNeill et al., 1988). Collectively, these data present a compelling argument for the potential of DBS-enhanced BDNF to mitigate nigrostriatal terminal dysfunction. Ultimately, future studies in which DBS is applied during the second, degenerative phase of the PFF model will be required to determine whether DBS-mediated effects on BDNF translate into neuroprotective effects.

**Figure 8.**
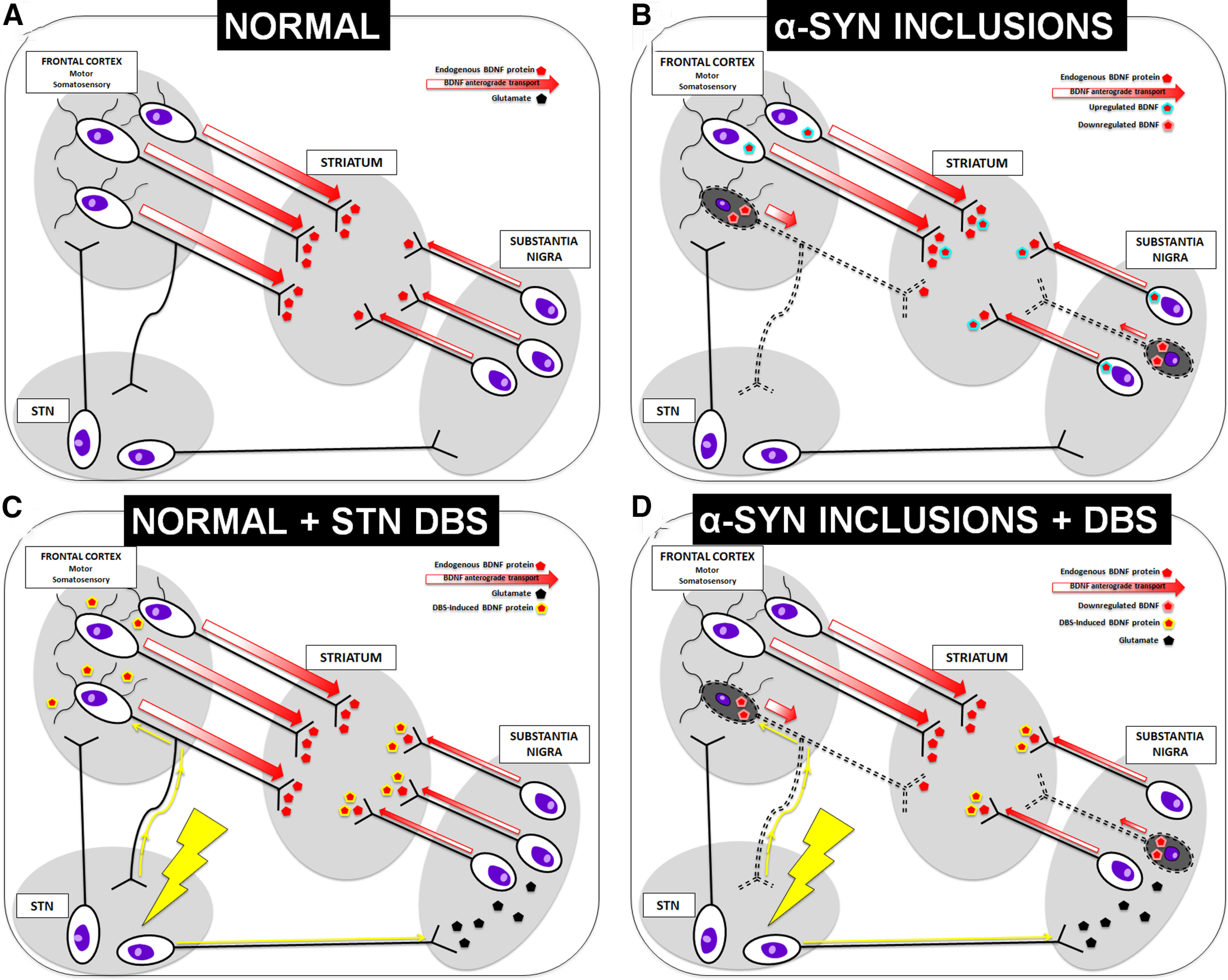
Schematic of possible mechanism. ***A***, Under normal conditions the majority of striatal BDNF is produced and transported from the frontal cortex, with some also coming from the substantia nigra (Altar and DiStefano, 1998). Note that some Layer V corticostriatal neurons also project to the STN (Kita and Kita, 2012). ***B***, Layer V corticostriatal neurons that project to the STN are in the region of cortex that accumulate pSyn inclusions (Paumier et al., 2015). α-Syn inclusions in corticostriatal and nigrostriatal neurons may limit BDNF anterograde transport. Non-inclusion bearing neurons may compensate for this decrease by an increase in BDNF production and release. ***C***, STN DBS in a naive animal results in increased BDNF protein in the cortex, substantia nigra, and striatum. Previous studies have shown that the DBS-induced striatal increase in BDNF is a result of increased release from the nigrostriatal system, rather than from the cortex (Spieles-Engemann et al., 2011). Antidromic activation of M1-STN circuitry may cause dendritic release of BDNF in the M1 cortex (Hartmann et al., 2001). ***D***, STN DBS applied to an inclusion-bearing rat results in elevated striatal BDNF protein, presumably from nigrostriatal neurons that have not been compromised by α-syn inclusions, albeit to a lesser degree than reported in naive rats (Spieles-Engemann et al., 2011). Importantly, M1 neurons that innervate the striatum also innervate the STN (Kita and Kita, 2012), suggesting that M1-STN neurons responsive to DBS may also have inclusions. Thus, the presence of inclusions in some corticostriatal neurons may impact the ability of STN DBS to drive an increase in M1 BDNF. Cortical BDNF protein is decreased, perhaps as a result of decreased production (because of inclusions) and lack of compensation, as a result of the nigral-driven striatal increase in BDNF.

The mechanism by which α-syn inclusions disrupt the normally positive relationship between corticostriatal and nigrostriatal BDNF levels remains unknown. Normally, the striatum does not produce its own BDNF; instead it is transported primarily from the cortex, and from the SNpc to lesser degree (Altar and DiStefano, 1998; schematic in Fig. 8*A*). BDNF protein levels in the cortex/nigra and striatum are positively correlated; that is, when cortical or nigral BDNF levels are high, striatal BDNF levels are also high (Fig. 6*E–G*). However, this association is eliminated when α-syn inclusions are present. BDNF mRNA is decreased in neurons with inclusions seeded by PFFs (Mahul-Mellier et al., 2020), suggesting that BDNF protein levels may also be decreased. However, we did not observe any impact of inclusions on total levels of BDNF protein in the M1 cortex, frontal cortex, substantia nigra, or striatum (Fig. 6*A–D*). Instead, only the relationship between corticostriatal and nigrostriatal BDNF was altered by inclusions. At the one-month time point, ∼30% of nigral neurons contain pSynir inclusions. Perhaps with a greater cortical inclusion load, cortical, nigral, and/or striatal BDNF protein levels would be reduced to such an extent that total BDNF levels within individual structures are impacted.

One possible explanation for why we observed an altered relationship between corticostriatal and nigrostriatal BDNF by inclusion status is that anterograde transport from the cortex (and nigra) to the striatum from inclusion-bearing neurons is impaired (Fig. 8*B*). *In vitro* evidence shows that impaired anterograde transport of the trkB BDNF receptor in inclusion-bearing primary neuronal cultures (Volpicelli-Daley et al., 2014), and impaired retrograde transport of BDNF itself in a α-syn overexpression model (Fang et al., 2017). As such, it is possible that pSyn inclusions result in a similar impairment of anterograde transport of BDNF itself. Importantly, in human PD (as in this model), only a subpopulation of nigral or cortical neurons have inclusions (Greffard et al., 2010; Milber et al., 2012). Thus, neighboring (inclusion free or reduced) neurons may compensate for decreased BDNF production by upregulating their BDNF production, transport, and release, resulting in a net zero change within a particular structure (Fig. 8*B*). Indeed, BDNF compensatory mechanisms have been observed in response to denervation of the nigrostriatal system, suggesting that a similar phenomenon could be occurring here (Yurek and Fletcher-Turner, 2000; Collier et al., 2005). Ultimately, additional studies will be required to definitively determine whether pSyn inclusion formation interferes with anterograde BDNF transport *in vivo*.

Similarly, we can speculate as to why STN DBS applied to a brain with pSyn accumulation may respond somewhat differently than a normal brain (Fig. 8*C*,*D*). Our previous studies indicate that the source of the DBS-induced striatal BDNF increase is the nigrostriatal system, based on lesion studies in which loss of nigrostriatal terminals shifts the DBS-induced increase from the striatum to the SN (Spieles-Engemann et al., 2011). We observe that nigrostriatal function is preserved enough to drive increased striatal BDNF when STN DBS is applied within an inclusion-bearing environment, albeit to a lesser magnitude than the naive brain (Spieles-Engemann et al., 2011). While the specific circuitry involved in DBS-induced M1 cortical BDNF elevation has not been identified, it is possible that antidromic activation of M1-STN circuitry causes dendritic release of BDNF in the M1 cortex (Hartmann et al., 2001). Importantly, M1 neurons that innervate the striatum also innervate the STN (Kita and Kita, 2012), suggesting that M1-STN neurons responsive to DBS may also have inclusions. Thus, the presence of inclusions in some corticostriatal neurons may impact the ability of STN DBS to drive an increase in M1 BDNF.

The disease-modifying potential of STN DBS remains unclear because of the fact that DBS has traditionally been a treatment of last resort. However, this practice may be shifting as several recent studies have examined the impact of earlier STN DBS, demonstrating significant therapeutic benefit (Schuepbach et al., 2013; Charles et al., 2014). For example, STN DBS in early PD has been shown to slow the progression of tremor (Hacker et al., 2015). Supporting the therapeutic potential of STN DBS, our results reveal novel effects of pSyn inclusions on BDNF handling. Specifically, inclusions prevent the normally positive association in BDNF protein levels in the nigrostriatal and corticostriatal circuits. Even in this context, STN DBS remains capable of increasing striatal BDNF. This finding suggests that DBS-induced striatal BDNF effects on nigrostriatal circuitry have the potential to modify the long-term neurodegenerative consequences of synucleinopathy.
